# Resource utilization and costs before and after total joint arthroplasty

**DOI:** 10.1186/1472-6963-12-73

**Published:** 2012-03-23

**Authors:** Kevin J Bozic, Brett Stacey, Ariel Berger, Alesia Sadosky, Gerry Oster

**Affiliations:** 1 University of California, San Francisco, Department of Orthopaedic Surgery, 500 Parnassus Ave. MUW320, San Francisco, CA 94143-0728, USA; 2 University of California, San Francisco, Philip R. Lee Institute for Health Policy Studies, 3333 California St., San Francisco, CA 94143-0936, USA; 3 Oregon Health & Science University, Portland, OR, USA; 4 Policy Analysis Inc., Brookline, MA, USA; 5 Pfizer Inc., New York, NY, USA

## Abstract

**Background:**

The purpose of this study was to compare pre- and post-surgical healthcare costs in commercially insured total joint arthroplasty (TJA) patients with osteoarthritis (OA) in the United States (U.S.).

**Methods:**

Using a large healthcare claims database, we identified patients over age 39 with hip or knee OA who underwent unilateral primary TJA (hip or knee) between 1/1/2006 and 9/30/2007. Utilization of healthcare services and costs were aggregated into three periods: 12 months "pre-surgery," 91 days "peri-operative," and 3 to 15 month "follow-up," Mean total pre-surgery costs were compared with follow-up costs using Wilcoxon signed-rank test.

**Results:**

14,912 patients met inclusion criteria for the study. The mean total number of outpatient visits declined from pre-surgery to follow-up (18.0 visits vs 17.1), while the percentage of patients hospitalized increased (from 7.5% to 9.8%) (both *p *< 0.01). Mean total costs during the follow-up period were 18% higher than during pre-surgery ($11,043 vs. $9,632, *p *< 0.01), largely due to an increase in the costs of inpatient care associated with hospital readmissions ($3,300 vs. $1,817, p < 0.01). Pharmacotherapy costs were similar for both periods ($2013 [follow-up] vs. $1922 [pre-surgery], p = 0.33); outpatient care costs were slightly lower in the follow-up period ($4338 vs. $4571, *p *< 0.01). Mean total costs for the peri-operative period were $36,553.

**Conclusions:**

Mean total utilization of outpatient healthcare services declined slightly in the first year following TJA (exclusive of the peri-operative period), while mean total healthcare costs increased during the same time period, largely due to increased costs associated with hospital readmissions. Further study is necessary to determine whether healthcare costs decrease in subsequent years.

## Background

Advanced osteoarthritis (OA) of the hip and knee is associated with severe pain, limitation in function, and impaired quality of life [1,2]. Patients with advanced OA of the hip or knee incur considerable healthcare costs related to pain medications (both oral and injectable), physical therapy, medical equipment, outpatient visits, and inpatient care [3]. In addition, the indirect or so-called "time costs" associated with lost productivity can be substantial [4]. Lower extremity (hip and knee) total joint arthroplasty (total knee arthroplasty [TKA], total hip arthroplasty [THA], collectively, TJA) has been associated with alleviation of pain, improvement in function and overall improvement in quality of life among patients with disabling arthritis of the hip and knee [5]. However, little is known about the economic impact of TJA on utilization of healthcare services and total cost of care after recovery from surgery in the United States. The purpose of this study was to compare utilization of healthcare services and direct healthcare costs in commercially insured TJA patients during the pre- and post-surgical periods.

## Methods

### Data source

Data were obtained from the PharMetrics Patient-Centric Database, which is comprised of facility, professional-service, and retail (i.e., outpatient) pharmacy claims from over 85 health plans throughout the United States. These plans provide health insurance to approximately 14 million people annually (Midwest, 35%; Northeast, 21%; South, 31%; West, 13%). All patient identifiers in the database are encrypted, and the database is fully compliant with the Health Insurance Portability and Accountability Act of 1996 (HIPAA). As no patient or provider contact was made and patient information was de-identified, Institutional Review Board (IRB) approval was not required.

Information available for each facility and professional-service claim includes date and place of service, diagnoses (in International Classifications of Diseases, 9^th ^Revision, Clinical Modification [ICD-9-CM] format), procedures (in ICD-9-CM [selected plans only], Current Procedural Terminology, 4^th ^Edition [CPT-4], and Healthcare Common Procedure Coding System [HCPCS] formats), provider specialty, and charged and paid amounts. Data available for each retail pharmacy claim include the drug dispensed (in National Drug Code [NDC] format), the dispensing date, and the quantity dispensed and number of days of therapy supplied (selected plans only). All claims include a charged amount; the database also provides paid (i.e., reimbursed, including patient deductible, copayment, and/or coinsurance) amounts.

Selected demographic, clinical, and eligibility information is also available, including age, gender, geographic region, primary and secondary diagnoses, coverage type, and the dates of insurance coverage. All patient-level data can be arrayed chronologically to provide a detailed, longitudinal profile of all medical and pharmacy services used by each plan member. The database for this study encompassed the period, January 1, 2005 through December 31, 2008 ("study period"), which represented the most recent period for which complete data were available at the point of study initiation.

### Study sample

The source population for this study consisted of all patients with any claims for unilateral primary TKR (CPT-4 code 27447 or ICD-9-CM procedure code 81.54) or unilateral primary THR (CPT-4 code 27130 or ICD-9-CM procedure code 81.51) between January 1, 2006 and September 30, 2007. (Patients undergoing partial or revision hip or knee replacements were excluded from the analysis). Among these patients, the date of the first-noted claim for TKR or THR was designated the "index procedure date", and patients not continuously enrolled in the database for the 12 month period prior to this date ("pre-surgery period") and the 15-month period subsequent to this date ("post-operative period") were excluded from the study sample.

Patients without diagnoses of OA (ICD-9-CM diagnosis codes 715.X5, 715.X6) in the three-month period preceding surgery were excluded from the study sample, as were those aged less than 40 years as of the time of their index procedure, those enrolled in Medicaid, and persons aged 65 years and older as of the time of their index procedure who were enrolled in a Medicare Advantage plan (because their claims histories are incomplete in the study database). Additional exclusions from the study sample included persons with evidence of any of the following during the one-year pre-surgery period: (1) prior TKR or THR; (2) joint fusion (CPT-4 27284, 27286, 27580); (3) procedures suggestive of a prior TKR/THR (CPT-4 codes 27090, 27091, 27134, 27137, 27138, 27488, 27486, 27487); (4) knee or hip fracture (ICD-9-CM diagnosis codes 820.XX, 821.2, 821.3, 823.0, 823.1) (in 30-day period prior to index procedure date only); (5) other conditions associated with TKR/THR (e.g., rheumatoid arthritis (ICD-9-CM codes 714, 714.X), post-traumatic arthritis (716.15, 716.16), avascular necrosis (733.42, 733.43) benign/malignant bone tumors (170.7, 213.7), or Paget's disease (731.0) during the pre-surgery period, and (6) patients who underwent a second primary TKA or THA during the 15-month period subsequent to the index procedure date^a^. All pharmacy, professional service, and facility claims were then compiled for all patients in the study sample over the one-year pre-surgery period and the 15-month post-operative period.

### Measures and analyses

The demographic and clinical characteristics of study subjects were examined, including prevalence of selected comorbidities, on the basis of information prior to the index procedure date. Patients were assumed to have a particular comorbidity if they had either one or more hospitalizations, or two or more outpatient claims at least 30 days apart, during the pre-surgery period with a corresponding diagnosis code/prescription.

Total healthcare costs were aggregated into three distinct periods: pre-surgery period (12-months prior to TJA); "peri-operative" (91-day period including and immediately following TJA); and "follow-up" (12-month period following the peri-operative period). Healthcare costs over each period were subcategorized into (1) pharmacotherapy (including oral, injectable, and topical agents); (2) outpatient care (including physician visits, other outpatient office visits [including but not necessarily limited to physical/occupational therapists), Emergency Department (ED) visits, and diagnostic imaging)]; (3) inpatient care; and (4) all other costs. Pharmacotherapy was further subcategorized as: (1) nonsteroidal anti-inflammatory drugs (NSAIDs), including cyclo-oxygenase (COX)-2 selective NSAIDs; (2) opioids, including tramadol; (3) antidepressants; (4) intra-articular corticosteroid and hyaluronic acid injections; and (5) all other medications.

Reimbursed amounts (i.e., payments made by third-party insurers as well as any patient liability [e.g., co-pays, co-insurance]) were used in all analyses of healthcare costs. All costs were expressed on an "as-is" basis (i.e., costs were not adjusted for inflation). Non-parametric bootstrapping (using sampling [with replacement] from the source population) was used to generate 95% confidence intervals (CIs) for all estimates of healthcare costs [6]. All estimates of cost were generated on a "per-patient" basis (e.g., total mean per-patient healthcare costs).

Mean total pre-surgery costs—for each sub-category and in aggregate—were compared with follow-up costs using Wilcoxon signed-rank tests; the percentages of patients with ≥1 hospitalizations during the pre-surgery versus follow-up periods were compared using McNemar's test. Utilization of healthcare services and costs occurring during the 90-day peri-operative period were excluded from analyses comparing pre-surgery with follow-up so as to compare healthcare costs and healthcare services utilization during the 1-year period prior to TJA with those of the 1-year period following recovery from TJA. (Levels of healthcare services utilization and costs during the peri-operative period were tabulated and reported separately.)

## Results

A total of 14,912 met the inclusion criteria for the study. The mean age of patients included in the study was 57.3 years, and 57% were women; approximately 17% of patients in our sample were 65 years of age or older (Table 1). All geographic regions of the country were represented, with the largest number of patients from the Midwest (41.9%), and the least number of patients from the West (13.5%). The most common comorbidities were hypertension (60.1%), hyperlipidemia (55.3%), and back pain (29.5%). Fifty-five percent of patients were enrolled in Preferred Provider Organizations (PPO), 24% were enrolled in Health Maintenance Organizations (HMO), and 16% were enrolled in Point of Service (POS) plans.

**Table 1:** Demographic and clinical characteristics of study subjects (N = 14,912)

Characteristic	Mean (%)
Mean Age (SD)	57.3 (7.3)

**Gender (N,%)**	

Male	6,417 (43.0%)

Female	8,495 (57.0%)

**Comorbidity**	

Hypertension	8,957 (60.1)

Hyperlipidemia	8,252 (55.3)

Neoplasms	4,522 (30.3)

Back pain	4,398 (29.5)

Painful neuropathic disorders	4,363 (29.3)

Chest pain	3,568 (23.9)

Diabetes	2,867 (19.2)

Fatigue	2,754(18.5)

Abdominal pain	2,678 (18)

Depressive disorders	2,160 (14.5)

Obesity	2,053 (13.8)

Sleep disorders	2,041 (13.7)

Ischemic heart disease	2,015 (13.5)

Cervical pain	1,687 (11.3)

Cardiac dysrythmias	1,612 (10.8)

Fibromyalgia	1,414 (9.5)

Anxiety disorders	1,302 (8.7)

Headache	1,298 (8.7)

Valve disease	1,158 (7.8)

Cerebrovascular disease	651 (4.4)

Migraine	530 (3.6)

Gout	479 (3.2)

Congestive heart failure	422 (2.8)

**Payer Type**	

HMO	3,546 (23.8)

PPO	8,169 (54.8)

POS	2,356 (15.8)

Other/unknown	622 (4.2)

**Census Region**	

Northeast	4,322 (29.0)

South	2,332 (15.6)

Midwest	6,250 (41.9)

West	2,008 (13.5)

Prescriptions for NSAIDs and opioids declined slightly during the follow-up period relative to the pre-surgery period (mean [SD] per-patient number of NSAID prescriptions = 2.0 [3.4] for pre-surgery vs 1.4 [2.9] for follow-up; for opioids, it was 2.5 [5.2] vs. 2.2 [5.4] [both *p *< 0.01]) (Table 2). Utilization of outpatient healthcare services, as measured by the number of outpatient visits, also declined slightly during the follow-up period. ED visits were unchanged between the two periods (mean [SD] per-patient value = 0.2 [0.7] for pre-surgery vs. 0.2 [0.9] for follow-up; *p *= 0.61)., Importantly, the percentage of patients with ≥1 hospitalizations increased (from 7.5% during pre-surgery to 9.8% during follow-up; *p *< 0.01).

**Table 2:** Levels of utilization of healthcare services during pre-surgery, peri-operative, and follow-up periods among study subjects (N = 14,912)*

	Period	Period	Period	
	**Pre-surgery**	**Peri-operative**	**Follow-up**	p-value**

**Prescriptions**				

**Pain-related**				

**Oral medications**				

NSAIDs^†^	2.0 (3.4)	0.4 (0.9)	1.4 (2.9)	< 0.01

Opioids	2.5 (5.2)	3.0 (3.1)	2.2 (5.4)	< 0.01

Antiepileptics	0.5 (2.2)	0.1 (0.6)	0.6 (2.6)	< 0.01

Antidepressants^‡^	1.6 (4.1)	0.4 (1.1)	1.9 (4.5)	< 0.01

**Injectable agents**				

Corticosteroids	0.9 (1.6)	0.1 (0.6)	0.5 (1.3)	< 0.01

Opioids	0.1 (0.6)	0.0 (0.3)	0.1 (0.6)	0.13

Hyaluronic acid	0.4 (1.5)	0.0 (0.1)	0.0 (0.4)	< 0.01

Total of above	1.4 (2.5)	0.1 (0.7)	0.6 (1.6)	< 0.01

**Topical agents**	0.1 (0.4)	0.0 (0.1)	0.1 (0.5)	< 0.01

**Total number of prescriptions**	7.1 (7.1)	9.2 (15.2)	10.7 (15.2)	< 0.01

**Healthcare services**				

**Outpatient care**				

Physician office visits	15.9 (13.3)	12.0 (9.7)	15.3 (15.0)	< 0.01

Other outpatient visits	3.2 (4.7)	2.7 (4.9)	2.6 (5.1)	< 0.01

ED visits	0.2 (0.7)	0.1 (0.4)	0.2 (0.9)	0.61

All outpatient visits	18.0 (14.3)	14.2 (9.6)	17.1 (16.1)	< 0.01

**Hospitalizations**				

No. (%) with ≥ 1 hospitalizations	1,115 (7.5)	14,392 (96.5)	1,456 (9.8)	< 0.01

*Unless otherwise noted, all values are per-patient mean (SD)

**Pre-surgery vs follow-up

†Includes acetaminophen

‡Comprised of tricyclic antidepressants, selective serotonin reuptake inhibitors, bupropion, buspirone, duloxetine, maprotiline, mirtazapine, nefazodone, and venlafaxine

Mean total per-patient healthcare costs during the follow-up period were 18% higher than during the pre-surgery period ($11,043 vs. $9,632, *p *< 0.01) (Table 3). Mean total per-patient costs for the 90-day peri-operative period (including those related to hospitalization for the index procedure) were $36,553. Per-patient pharmacotherapy costs were similar for both the follow-up and pre-surgery periods ($2013 [follow-up] vs. $1922 [pre-surgery], *p *= 0.33).

**Table 3:** Mean per-patient healthcare costs during pre-surgery, peri-operative, and follow-up periods among study subjects (N = 14,912)*

	Period			
	
	Pre-surgery	Peri-operative	Follow-up	p-value**
**Prescriptions**				

**Pain-related**				

**Oral medications**				

*NSAIDs^†^*	153 (147, 158)	28 (26, 29)	98 (93, 103)	< 0.01

*Opioids*	97 (79, 116)	60 (56, 64)	110 (83, 137)	< 0.01

*Antiepileptics*	58 (52, 64)	15 (13, 17)	77 (66, 87)	< 0.01

*Antidepressants*^‡^	146 (138, 154)	35 (33, 37)	154 (146, 162)	< 0.01

*Corticosteroids*	2 (2, 2)	0 (0, 1)	2 (2, 3)	0.16

**Injectable agents**				

*Corticosteroids*	15 (12, 18)	4 (2, 6)	10 (6, 13)	< 0.01

*Opioids*	2 (2, 2)	1 (1, 1)	2 (2, 2)	0.09

*Hyaluronic acid*	90 (84, 95)	1 (1, 1)	6 (5, 8)	< 0.01

*Total of above*	107 (101, 113)	6 (4, 8)	18 (15, 22)	< 0.01

**Topical agents**	2 (1, 2)	0 (0, 1)	4 (3, 5)	< 0.01

**All other**	1,358 (1,318, 1,389)	459 (447, 471)	1,550 (1,506, 1,594)	< 0.01

**Total Pharmacotherapy**	1,922 (1,870, 1,974)	604 (589, 619)	2,013 (1,954, 2,072)	0.33

**Healthcare services**				

**Outpatient care**				

**Physician office visits**	2,364 (2,320, 2,408)	1,410 (1,383, 1,437)	2,148 (2,096, 2,199)	< 0.01

**Other outpatient visits**	2, 024 (1,901, 2,148)	1,978 (1,844, 2,111)	1,980 (1,812, 2,148)	< 0.01

**ED visits**	183 (169, 197)	96 (87, 105)	211 (195, 227)	0.06

**All outpatient visits**	4,571 (4,429, 4,714)	3,484 (3,345, 3,622)	4,338 (4,149, 4,528)	< 0.01

**Inpatient care**	1,817 (1,655, 1,979)	31,116 (30,802, 31,431)	3,300 (3,007, 3,593)	< 0.01

**Other**				

OA-related^¥^	30 (27, 33)	48 (46, 50)	13 (11, 15)	< 0.01

All other	1,293 (1,229, 1,356)	1,300 (1,248, 1,353)	1,379 (1,297, 1,461)	< 0.01

**Total**	9,632 (9,364, 9,901)	36,553 (36,221, 36,885)	11,043 (10,628, 11,458)	< 0.01

*Unless otherwise noted, all values are mean (95% confidence interval) per-patient healthcare costs ($)

**Pre-surgery vs follow-up

†Includes acetaminophen

‡Comprised of tricyclic antidepressants, selective serotonin reuptake inhibitors, bupropion, buspirone, duloxetine, maprotiline, mirtazapine, nefazodone, and venlafaxine

¥Medical equipment

However, within pharmacotherapy, mean total per-patient follow-up costs were lower than mean total pre-surgery costs for NSAIDs ($98 vs. $15, *p *< 0.01) and injectable agents ($18 vs. $107, *p *< 0.01), but higher for opioids ($110 vs. $97, *p *< 0.01), antidepressants ($154 vs. $146, *p *< 0.01), and all other medications ($1550 vs. $1358, *p *< 0.01). On a per-patient basis, outpatient care costs were slightly lower in the follow-up period than in the pre-surgery period ($4338 vs. $4571, *p *< 0.01), whereas mean per-patient inpatient costs were substantially higher in the follow-up period when compared with the pre-surgery period ($3300 vs. $1817, *p *< 0.01). All other per-patient healthcare costs were also slightly higher in the follow-up period ($1392 vs. $1323, *p *< 0.01).

## Discussion

The economic burden of osteoarthritis has been increasing in the United States, in part due to an increase in the prevalence of disease, and to increasing costs of treatment (both surgical and non-surgical) [7,8]. At the same time, concerns have been raised regarding the costs associated with TJA, due to an increase in the number of procedures performed annually in the U.S., management of post-surgical complications, and increased adoption of newer, more expensive implant technologies [9]. TJA has been shown to be a cost-effective intervention relative to non-operative treatment [10-13] and has been associated with dramatic improvements in quality of life in patients who suffer from debilitating OA of the hip and knee [5,14,15]. Several investigators have shown that when performed in younger patients, TJA can expedite return to work and a more active, productive lifestyle [16,17].

While previous authors have reported that OA is associated with substantial direct and indirect medical expenditures [6,16,17], relatively little is known about the direct medical costs before and after hip and knee arthroplasty in patients with OA. Hawker and colleagues undertook a nested case-control study to examine total and arthritis-attributable costs in the periods before and after TJA among persons with disabling arthritis of the hip and/or knee in Ontario, Canada [18]. The pre-surgery period was defined as the 1-year period ending 6 weeks prior to TJA; the peri-operative period, as beginning 6 weeks prior to TJA and ending 6 months following TJA; and the post-surgery period, the 1-year period thereafter. The authors found that while mean arthritis-attributable costs during post-surgery decreased from pre-surgery levels by $278 (Canadian dollars, expressed in calendar-year [CY] costs), total healthcare costs increased by $1978. In their analyses of total healthcare costs of patients who underwent TJA for any reason (exclusive of bone cancers) during the 3-year periods immediately before and after surgery, Graver et al. [19] reported total healthcare costs of $8762, $10,076, and $11,475 in the third, second, and year immediately prior to TJA, respectively; corresponding values for the 3 years immediately following and including the surgery were $37,445, $11,980, and $11,307. While Graver and colleagues did not account for the peri-operative period, which render direct comparisons between their work and ours somewhat difficult, we note that their reported costs in the second year subsequent to TJA are higher than those reported in the year prior to surgery (statistical significance testing not undertaken).

Our results indicate that although utilization of healthcare services decreased for most services during the first year following TJA (exclusive of the peri-operative period) in commercially insured patients with OA of the hip or knee, mean total healthcare costs increased, largely due to increased costs associated with inpatient care. Given that the mean length of stay during the peri-operative period (a 3-month "window" that includes the admission for the initial TJA as well as any subsequent readmission) was only 6.9 days, and that 75% of patients spent no more than 8 days in hospital during this time, our findings are most likely associated with readmissions, rather than stays in hospital for the initial TJA of > 91 days. Hospital readmissions following elective surgical procedures such as TJA have become the focus of great debate and concern among healthcare policymakers [20], and have led to calls for changes in payment policy to incentivize better coordination of post-discharge care among providers [21,22]. Based on our findings, further investigation is warranted into the cause of and strategies to decrease the rate of hospital readmissions following elective TJA.

Despite the interesting findings, our study has several limitations. First, our database consisted of administrative claims for patients enrolled in private insurance plans, who tend on average to be younger than the "typical" cohort who undergoes TJA for OA. Specifically, while patients aged ≥65 years comprise approximately two-thirds of all patients who undergo TJA in the US [23], they only represented about 17% of patients in our sample. However, we would note that as the indications for TJA have expanded to include younger, more active patients, an increasing number of patients under the age of 65 are undergoing TJA [24].

Second, our analysis relied upon administrative claims data, which have been shown to have an imperfect correlation with the clinical record [25]. It is possible that certain costs may have been over- or under-estimated in both the pre-surgery and follow-up period due to the reliance on administrative claims data. However, we believe that the majority of healthcare costs experienced by patients in the periods immediately before and after TJA are captured within the database. Because the principal purpose of administrative databases such as the one we used in this study are to support reimbursement, we believe that our study yields a fairly comprehensive view of "real-world" utilization and cost of healthcare services in commercially insured patients who undergo TJA.

Third, and somewhat related to the second, we did not attempt to adjust for price inflation by expressing costs in terms of a particular "base year". The principal reason that we did not undertake such an adjustment was that we thought it important to examine (and report) actual amounts paid in "real-word" clinical practice during the periods before and after joint replacement. Were we to have expressed all costs in terms of a "base year", such information would not be available. We also note that identification of an appropriate "price index" to adjust healthcare costs is not straightforward. In most instances where this method is employed, a single index (e.g., the medical-care component of the US consumer price index) is used to adjust values; implicit in use of this single index, however, is the assumption that all goods and services (e.g., pharmacotherapy, physician office visits, hospitalizations, durable medical equipment) experience the same relative change in pricing in a given year. The degree to which that assumption is valid within any given year is unknown. Regardless, to the extent that differences between pretreatment and follow-up costs reflect price inflation, our findings may overestimate differences in healthcare costs between these two periods.

Fourth, although recovery from TJA surgery is variable, it is possible that some patients were not fully recovered from surgery by 90 days [26], and therefore some of the costs included in the follow-up period may have been related to recovery from TJA. The 90-day window for the peri-operative period chosen because this is considered the 'global period' by most insurers, during which time all costs that are incurred are considered attributable to the index procedure. This limitation is further mitigated by the fact that the major difference in costs between the pre-surgery and follow-up period was related to inpatient care, and it is unlikely that these costs would be considered routine follow-up costs. Since our goal was to evaluate the impact of TJA on healthcare resource utilization and total direct healthcare spending during the first 15 months following surgery from a healthcare system perspective, we did not attempt to separate out costs that were directly attributable to OA or TJA, which may account for the difference in our findings from those of Hawker, et al. [18] Additionally, we were unable to include indirect or time costs in our analysis, and it is quite possible that the increase in direct medical costs during the follow-up period could be offset by reductions in indirect or time costs associated with earlier return to work and increased productivity, especially given the relatively young average age (57.3 years) of the commercially insured patients who were included in our study. Additionally, we only measured costs during the 15 months immediately following the index TJA procedure, while the benefits of TJA are known to accrue over many years and in some cases decades. Nonetheless, it is anticipated that most patients (especially the younger patients included in this study) recover completely from surgery within 3 months, and therefore, their use of healthcare services and the associated costs would be expected to decrease after that time.

## Conclusions

In summary, despite slightly lower outpatient care costs and similar costs associated with pharmacotherapy, we found that mean total direct health care costs increased during the 12-month period following recovery from TJA when compared with the 12-month period prior to surgery in commercially insured patients with OA, in large part due to increased costs associated with hospital readmissions. Further study is necessary to determine whether healthcare costs decrease in subsequent years.

## Endnotes

^a^Patients who underwent a second primary TKA or THA during the 15-month period subsequent to the index procedure were excluded, as these procedures are often scheduled electively following recovery from the index procedure, they are not indicative or reflective of the condition of the index joint, and they substantially impact the cost and utilization of healthcare services during the time period under study.

## Competing interests

This study was funded by Pfizer Inc. Alesia Sadosky is a full-time employee of Pfizer Inc. and owns stock in the company. The analyses were conducted by Ariel Berger and Gerry Oster, employees of Policy Analysis, Inc. Policy Analysis, Inc. received financial support from Pfizer, Inc. for the conduct of this analysis and development of this manuscript. Drs. Kevin Bozic and Brett Stacey did not receive any financial support from Pfizer. The funding source did not play a role in the investigation or the interpretation of the results.

## Authors' contributions

KB, BS, AB, AS, GO designed the study. AB and AS gathered the data. KB, AB, AS, analyzed the data. KB, BS, AB, AS, GO wrote the initial draft. KB, BS, AB, AS, GO ensured the accuracy of the data and analysis. All authors read and approved the final manuscript.

## Pre-publication history

The pre-publication history for this paper can be accessed here:

http://www.biomedcentral.com/1472-6963/12/73/prepub

## References

[B1] Felson DT Clinical practice. Osteoarthritis of the knee N Engl J Med 2006 354 8 841 8 10.1056/NEJMcp051726 16495396

[B2] Lane NE Clinical practice. Osteoarthritis of the hip N Engl J Med 2007 357 14 1413 21 10.1056/NEJMcp071112 17914042

[B3] Loza E Economic burden of knee and hip osteoarthritis in Spain Arthritis Rheum 2009 61 2 158 65 10.1002/art.24214 19177521

[B4] Gabriel SE Indirect and nonmedical costs among people with rheumatoid arthritis and osteoarthritis compared with nonarthritic controls J Rheumatol 1997 24 1 43 8 9002009

[B5] Laupacis A The effect of elective total hip replacement on health-related quality of life J Bone Joint Surg Am 1993 75 11 1619 26 8245054 10.2106/00004623-199311000-00006

[B6] Efron B Tibshirani R Bootstrap methods for standard errors, confidence intervals, and other measures of statistical accuracy Stat Sci 1986 1 54 77 10.1214/ss/1177013815

[B7] Bitton R The economic burden of osteoarthritis Am J Manag Care 2009 15 8 Suppl S230 5 19817509

[B8] Yelin E Callahan LF The economic cost and social and psychological impact of musculoskeletal conditions. National Arthritis Data Work Groups Arthritis Rheum 1995 38 10 1351 62 10.1002/art.1780381002 7575685

[B9] Bozic KJ Economic evaluation in total hip arthroplasty: analysis and review of the literature J Arthroplasty 2004 19 2 180 9 10.1016/S0883-5403(03)00456-X 14973861

[B10] Katz JN Total joint replacement in osteoarthritis Best Pract Res Clin Rheumatol 2006 20 1 145 53 10.1016/j.berh.2005.09.003 16483913

[B11] Rasanen P Effectiveness of hip or knee replacement surgery in terms of quality-adjusted life years and costs Acta Orthop 2007 78 1 108 15 10.1080/17453670610013501 17453401

[B12] Losina E Cost-effectiveness of total knee arthroplasty in the United States: patient risk and hospital volume Arch Intern Med 2009 169 12 1113 21 discussion 1121-2 10.1001/archinternmed.2009.136 19546411 PMC2731300

[B13] Xie F Total or partial knee replacement? Cost-utility analysis in patients with knee osteoarthritis based on a 2-year observational study Eur J Health Econ 2010 11 1 27 34 10.1007/s10198-009-0154-5 19430952

[B14] Soderman P Outcome after total hip arthroplasty: Part II. Disease-specific follow-up and the Swedish National total hip arthroplasty register Acta Orthop Scand 2001 72 2 113 9 10.1080/000164701317323345 11372940

[B15] Cushnaghan J Long-term outcome following total knee arthroplasty: a controlled longitudinal study Ann Rheum Dis 2009 68 5 642 7 10.1136/ard.2008.093229 18664545

[B16] Diduch DR Total knee replacement in young, active patients. Long-term follow-up and functional outcome J Bone Joint Surg Am 1997 79 4 575 82 9111404 10.2106/00004623-199704000-00015

[B17] Mobasheri R Gidwani S Rosson JW The effect of total hip replacement on the employment status of patients under the age of 60 years Ann R Coll Surg Engl 2006 88 2 131 3 10.1308/003588406X95129 16551399 PMC1964053

[B18] Hawker GA A population-based nested case-control study of the costs of hip and knee replacement surgery Med Care 2009 47 7 732 41 10.1097/MLR.0b013e3181934553 19536034

[B19] Graver RM The Total "Economic Cost" of primary total joint replacement surgery in the United States Poster 272 presented at: 2010 Annual Meeting of American Academy of Orthopaedic Surgeons 2010 New Orleans, LA *CeraNews. The Orthopaedic Information Landscape Journal *2010, 2:6

[B20] Minott J Reducing Hospital Readmissions Academy Health 2008

[B21] Birenbaum S Program to reduce preventable hospital readmissions launches in California California Healthcare Foundation 2010

[B22] Medicare Payment Advisory Commission (MedPAC) Report to the Congress: Medicare Payment Policy 2010 10.3109/15360288.2010.503732 20718654

[B23] Kim S Changes in surgical loads and economic burden of hip and knee replacements in the US: 1997-2004 Arthritis Rheum 2008 59 4 481 8 10.1002/art.23525 18383407

[B24] Nunley RM Do patients return to work after hip arthroplasty surgery J Arthroplast 2011 26 6 Suppl 92 98 e1-3 10.1016/j.arth.2011.03.038 21602024

[B25] Bozic KJ The validity of using administrative claims data in total joint arthroplasty outcomes research J Arthroplast 2010 25 6 Suppl 58 61 10.1016/j.arth.2010.04.006 20570479

[B26] Johnston L The Knee Arthroplasty Trial (KAT) design features, baseline characteristics, and two-year functional outcomes after alternative approaches to knee replacement J Bone Joint Surg Am 2009 91 1 134 41 19122088 10.2106/JBJS.G.01074

